# Including dominance effects in the prediction model through locus-specific weights on heterozygous genotypes can greatly improve genomic predictive abilities

**DOI:** 10.1038/s41437-022-00504-6

**Published:** 2022-02-05

**Authors:** Tianfei Liu, Chenglong Luo, Jie Ma, Yan Wang, Dingming Shu, Hao Qu, Guosheng Su

**Affiliations:** 1 State Key Laboratory of Livestock and Poultry Breeding, Institute of Animal Science, Guangdong Academy of Agricultural Sciences, Guangzhou, 510640 China; 2 Guangdong Provincial Key Laboratory of Animal Breeding and Nutrition, Institute of Animal Science, Guangdong Academy of Agricultural Sciences, Guangzhou, 510640 China; 3 Center for Quantitative Genetics and Genomics, Department of Molecular Biology and Genetics, Aarhus University, DK-8830 Tjele, Denmark

**Keywords:** Animal breeding, Quantitative trait

## Abstract

The dominance effect is considered to be a key factor affecting complex traits. However, previous studies have shown that the improvement of the model, including the dominance effect, is usually less than 1%. This study proposes a novel genomic prediction method called CADM, which combines additive and dominance genetic effects through locus-specific weights on heterozygous genotypes. To the best of our knowledge, this is the first study of weighting dominance effects for genomic prediction. This method was applied to the analysis of chicken (511 birds) and pig (3534 animals) datasets. A 5-fold cross-validation method was used to evaluate the genomic predictive ability. The CADM model was compared with typical models considering additive and dominance genetic effects (ADM) and the model considering only additive genetic effects (AM). Based on the chicken data, using the CADM model, the genomic predictive abilities were improved for all three traits (body weight at 12th week, eviscerating percentage, and breast muscle percentage), and the average improvement in prediction accuracy was 27.1% compared with the AM model, while the ADM model was not better than the AM model. Based on the pig data, the CADM model increased the genomic predictive ability for all the three pig traits (trait names are masked, here designated as T1, T2, and T3), with an average increase of 26.3%, and the ADM model did not improve, or even slightly decreased, compared with the AM model. The results indicate that dominant genetic variation is one of the important sources of phenotypic variation, and the novel prediction model significantly improves the accuracy of genomic prediction.

## Introduction

With the continuous growth of the global population, human demand for food and security continues to increase. Hybridization is an important tool for increasing meat and grain production. It has been successfully applied to the production of animals (such as chickens and pigs) and plants (such as corn and rice). The performance of hybrid individuals is affected by genetic factors such as additive, dominance, and epistatic genetic effects. Traditional genetic evaluation methods only consider additive genetic effects but not nonadditive genetic effects (dominance and epistatic effects). Many studies have shown that nonadditive genetic effects are an important component of phenotypic values (Costa-Neto et al. 2021; Mao et al. 2020; Su et al. 2012; Vitezica et al. 2017; Wang et al. 2020; Xu 2013). Li et al. (2017) reported that the dominant variance of broiler feed-related traits accounted for 29.5–58.4% of the genetic variance. There are three main reasons why traditional genetic evaluation methods only consider additive effects. (1) Additive genetic effects are the additive effects of a number of genes that can be accumulative and stably inherited, while nonadditive genetic effects are the effects of gene interactions that can change by allele recombination and thus cannot be stably inherited. (2) The traditional pedigree-based genetic evaluation method has difficulty accurately estimating nonadditive genetic effects since pedigree-based nonadditive genetic relationships between individuals are usually weak, and it is difficult to distinguish nonadditive genetic effects (such as dominance effects) and common environmental effects on siblings. (3) There is a high computational demand for computing the inverses of relationship matrices for non-additive genetic effects for large data.

Genomic prediction may overcome the limitation of conventional pedigree-based prediction in the estimation of nonadditive genetic effects. Because genomic prediction is based on genome information to predict the genetic value of individuals, it can provide a new approach to detect nonadditive genetic relationships between animals in the population and accurately distinguish the genotypic differences between sibling individuals (Meuwissen et al. 2001; Su et al. 2012). There is increasing interest in studies on genomic prediction considering nonadditive genetic effects, especially dominance effects (Su et al. 2012; Vitezica et al. 2017; Wang et al. 2020; Xiang et al. 2018). Genomic prediction models fitting dominance effects have been developed and constantly improved. Su et al. (2012) proposed a genomic model that fits the dominance effect at the unweighted scale of the dominance effect. Vitezica et al. (2013) presented a model fitting dominance effect on the scale of the gene substitution effect, which is consistent with the classical definition of quantitative genetics and breeding. Furthermore, Vitezica et al. (2017) proposed an expanded method from the natural and orthogonal interactions (NOIA) approach, which uses genotypic (not allelic) frequencies to construct incidence matrix. Xiang et al. (2018) proposed a model with a covariance matrix to account for the relationship between additive and dominance effects. However, using these models, many studies have reported that the improvement in accuracy of predicting genetic values is very small, mostly less than 1%, or even no improvement (Aliloo et al. 2016; Su et al. 2012; Vitezica et al. 2013; Xiang et al. 2018).

Although many studies have shown that models considering dominance effects do not lead to a clear improvement in predicting breeding values and total genetic values, many studies have shown that dominance variances are considerable in complex traits of animals and plants (Su et al. 2012; Vitezica et al. 2017; Xu et al. 2014). The universal phenomenon of heterosis in animals and plants also indicates that dominance effects are substantial. Therefore, there is a need for alternative statistical methods and models that can efficiently estimate genetic values, including dominance effects. This study proposed a novel method to account for genomic dominance effects, which integrated additive and dominance effects through locus-specific weights on heterozygous genotypes to construct a genomic relationship matrix. The model was tested by validation of prediction accuracy based on chicken and pig data.

## Materials and methods

### The novel prediction model

The model developed in this study is based on the assumption that the degree of dominance is different at different loci, and thus, the model accounts for the difference in degrees of dominance by locus-specific weights on heterozygous genotypes. The approach includes two main steps: (1) estimating the dominant degree based on the deviation between the heterozygous and homozygous marker genotypes; and (2) using the estimated degree of dominance as weights on heterozygous genotypes to construct a genomic relationship matrix. The details of the approach are as follows.

Let the A_1_ homozygous (A_1_A_1_), heterozygous (A_1_A_2_), and A_2_ homozygous (A_2_A_2_) genotypes be coded as 0, 1, and 2, respectively. The prior degree of dominance at a locus is defined as

$$x_{min} = {{{\mathrm{min}}}}\left( {\overline x _{A_1A_1},\overline x _{A_2A_2}} \right),$$

$$x_{max} = {{{\mathrm{max}}}}\left( {\overline x _{A_1A_1},\overline x _{A_2A_2}} \right),$$

$$d = 2 \ast \left( {\overline x _{A_1A_2} - x_{min}} \right)/\left( {x_{max} - x_{min}} \right)$$

where $\overline x _{A_1A_1}$, $\overline x _{A_1A_2}$ and $\overline x _{A_2A_2}$ are the means of corrected phenotypes of the three genotypes A_1_A_1_, A_1_A_2_, and A_2_A_2_, respectively.

The weighted heterozygous genotype (C_d_) is defined as:

$$C_d = \left\{ {\begin{array}{*{20}{c}} {0,\,if\,d \le 0,} \\ {d,\,if\,0\, < \,d\, < \,2} \\ {2,\,if\,d \ge 2.} \end{array},} \right.$$

At the same time, if $\overline x _{A_1A_1}\, > \,\overline x _{A_2A_2}$, A_1_A_1_ and A_2_A_2_ are recoded as 2 and 0, respectively.

The boundaries of 0 and 2 for *C*_d_ are to restrict the degree of dominance in the range from zero to complete dominance. Relaxation of the boundaries will allow overdominance.

The above modified genotype coefficients are then applied to construct a genomic relationship matrix in the best linear unbiased predictor (GBLUP) model for estimating the genetic values, including additive and dominance effects. The genomic relationship matrix can be built using the same method as those for the additive genomic relationship matrix (e.g., VanRaden 2008), applying a genotype matrix built with *C*_d_ values. A simple simulation in R language was shown to illustrate the calculation of genotype matrix (Supplementary File 1).

### Data

The novel model was tested using two published datasets from chickens (Liu et al. 2014) and pigs (Cleveland et al. 2012). The chicken dataset contained 511 F_2_ birds from two outbred lines. All birds had phenotypic records. The phenotypes were body weight at the 12th week (W12), eviscerating percentage (EP), which is percentage of eviscerated weight to body weight before slaughter, and breast muscle percentage (BMP), which is percentage of breast muscle weight to eviscerated weight. The phenotypes in the chicken data (from one herd) were corrected for fixed effects (sex and batch), and the fixed effects were estimated using linear least squares approach by linear fixed effects model. The birds were genotyped using the Illumina Chicken 60K SNP BeadChip. SNP markers with a minor allele frequency (MAF) lower than 0.01 were deleted. After editing, 46,672 SNPs were used to build the relationship matrix.

The pig dataset consisted of 3534 animals from a single PIC nucleus pig line. The data concealed the actual trait names but used the symbols T1, T2, T3, T4, and T5 instead. Three traits (T1, T2, and T3) were analyzed in this study, and the traits had 2804, 2715, and 3141 phenotypic records, respectively. The three traits were either corrected for environmental factors (e.g., year of birth or farm) and rescaled by correcting for the overall mean (T3) or was a rescaled, weighted mean of corrected progeny phenotypes (T1 and T2), for which many pigs have no individual phenotype (Cleveland et al. 2012). All 3534 animals were genotyped with the Illumina PorcineSNP60 BeadChip. SNP markers with a MAF lower than 0.01 were also deleted. After editing, 52,842 SNPs were used for calculating the relationship matrix.

### Statistical analysis

The data were analyzed using three statistical models, i.e., the novel model proposed in this study, a typical GBLUP model considering additive genetic effects only (VanRaden 2008), and a typical additive and dominance GBLUP model (Su et al. 2012).

### Genomic BLUP model combining additive and dominance genetic effects

The novel model proposed in this study (CADM) integrates additive and dominance genetic effects and can be written as:

$${{{\mathbf{y}}}} = {\boldsymbol{1}}{{\upmu}} + {{{\boldsymbol{Z}}}}_{{{\boldsymbol{g}}}}{{{\mathbf{g}}}} + {{{\mathbf{e}}}},$$

where **y** is the vector of phenotypic trait records, μ is the overall mean, **1** is a vector of 1s, **Z**_g_ is incidence matrix of **g**, **g** is the vector of the genotype value combining additive and dominance effects, and **e** is the vector of random residuals. It is assumed that **g** ~ N(**0**, ${{{\mathbf{G}}}}_{{{{\boldsymbol{ad}}}}}\sigma _g^2$) and **e** ~ N(**0**, ${{{\mathbf{I}}}}\sigma _e^2$), where **G**_ad_ is the additive-dominance genomic relationship matrix, **I** is an identity matrix, $\sigma _g^2$ is the additive-dominance genetic variance, and $\sigma _e^2$ is the residual variance.

The combined additive and dominance genomic relationship matrix **G**_**ad**_ was constructed using the modified marker genotype coefficients (C_d_), where locus-specific weights were exerted on heterozygous genotypes, and a method similar to the additive genomic relationship matrix was applied (VanRaden 2008). Briefly, ${{{\mathbf{G}}}}_{{{{\boldsymbol{ad}}}}} = \frac{{{{{\mathbf{M}}}}_{{{{\boldsymbol{ad}}}}}{{{\mathbf{M}}}}_{{{{\boldsymbol{ad}}}}}^\prime }}{{{\sum} {2p_i\left( {1 - p_i} \right)} }}$, where **M**_**ad**_ is a (*n* × *m*) matrix (*n* = number of individuals, *m* = number of marker loci) that specifies SNP genotype coefficients at each locus. The coefficients of the *i*^th^ column in matrix **M**_**ad**_ are (0–2*p*_*i*_) for A_1_A_1_, (C_d_–2*p*_*i*_) for A_1_A_2_ where C_d_ was as defined earlier, and (2–2*p*_*i*_) for A_2_A_2_, where *p*_*i*_ is the frequency of allele 1 (A_1_) at locus *i*.

CADM can be considered as a (co)variance matrix, but the definition is not exactly the same as the one in conventional breeding model. In CADM, we considered dominance effect as 0 for homozygous and non-zero for heterozygous, i.e., a genomic model (Su et al. 2012; Vitezica et al. 2013).

### Genomic BLUP model for additive genetic effects

The linear mixed model with additive genetic effects (AM) is:

$${{{\mathbf{y}}}} = {\boldsymbol{1}}{{\upmu}} + {{{\boldsymbol{Z}}}}_{{{\boldsymbol{a}}}}{{{\mathbf{a}}}} + {{{\mathbf{e}}}},$$

where the definitions of **y** and **e** are the same as those in the CADM model, where **a** is the vector of additive genetic effects, **Z**_**a**_ is the corresponding incidence matrix. It was assumed that **a** ~ *N* (**0**, ${{{\mathbf{G}}}}\sigma _a^2$), where **G** is the additive relationship matrix and $\sigma _a^2$ is the additive genetic variance.

The additive genomic relationship matrix **G** was constructed using the original SNP marker information (VanRaden 2008), in which elements of **M** are coded as (0–2*p*_*i*_) for A_1_A_1_, (1–2*p*_*i*_) for A_1_A_2_, and (2–2*p*_*i*_) for A_2_A_2_ at locus *i*.

### Genomic BLUP model including additive and dominance genetic effects as two components

The linear mixed model including additive and dominance genetic effects (ADM) is:

$${{{\mathbf{y}}}} = {\boldsymbol{1}}{{{{\upmu }}}} + {{{\boldsymbol{Z}}}}_{{{\boldsymbol{a}}}}{{{\mathbf{a}}}} + {{{\boldsymbol{Z}}}}_{{{\boldsymbol{d}}}}{{{\mathbf{d}}}} + {{{\mathbf{e}}}},$$

where **d** is the vector of dominance effects, **Z**_**d**_ is the corresponding incidence matrix. It is assumed that **d** ~ *N* (**0**, ${{{\mathbf{D}}}}\sigma _d^2$), where **D** is the dominance relationship matrix, and $\sigma _d^2$ is the dominance genetic variance.

The method for constructing the additive genomic relationship matrix **G** is the same as in the AM model. The dominance genomic relationship matrix **D** is constructed by ${{{\mathbf{D}}}} = \frac{{{{{\mathbf{HH}}}}^\prime }}{{{\sum} {\left( {2p_iq_i} \right)^2} }}$ (Vitezica et al. 2013) where **H** is a (*n* × *m*) matrix of heterozygosity coefficients. The coefficients of the *i*^*th*^ column in matrix **H** are (−2*p*_*i*_^2^) for A_1_A_1_, (2*p*_*i*_*q*_*i*_) for A_1_A_2_, and (−2*q*_*i*_^2^) for A_2_A_2_, where *q*_*i*_ and *p*_*i*_ are the frequencies of allele 1 (A_1_) and allele 2 (A_2_) at locus *i*, respectively.

The variance-covariance components were estimated using the average information restricted maximum likelihood (AIREML) (Gilmour et al. 1995). The estimation of variance components and the prediction of genetic effects were carried out using the DMU package (Madsen et al. 2010).

### Cross-validation

The accuracy of genomic prediction was verified by five-fold cross-validation. The entire dataset was randomly split into five subsets of similar size. In each fold of cross-validation, one of the five subsets was used as the validation data in which the phenotypic values were masked during genomic prediction; the remaining four subsets were used as the training data. For each validation, the data used to obtain weights on heterozygous genotypes in the CADM method were in line with the training population (i.e., the records in the validation population were excluded from the data to calculate the degree of dominance). The cross-validation was repeated 10 times.

Prediction accuracy was defined as the correlation between the predictions and corrected phenotypic values. Differences between the correlations from different models were tested by bootstrapping (Efron 1979) and Bonferroni’s method (Bland and Altman 1995). Bootstrap and Bonferroni’s methods were performed with the sample() function and “agricolae” package in R (R Core Team 2020).

## Results

### Genetic variances

Table 1 shows the variances in proportion to the phenotypic variance for the three quantitative traits (BMP, EP, and W12) from the chicken dataset. For W12, the composite genetic variance (composites of additive and dominance genetic variances) estimated from the CADM model was lowest, and the total genetic variance estimated from the ADM model was highest among the three models. The additive genetic variance estimated from the ADM model was lower than that from the AM model. For EP, the composite genetic variance estimated from the CADM model was similar to the total genetic variance estimated from the ADM model, and the additive genetic variance estimated from the ADM model was lower than that from the AM model. For BMP, the composite genetic variance estimated from the CADM model was highest, the ADM model did not capture dominance genetic variance, and the additive genetic variance was consistent with that from the AM model.

**Table 1:** Proportions of genetic variance components to phenotypic variance based on the three genomic models^a^ for the chicken dataset^b^.

Trait	CADM	ADM			AM
	V_G*_/V_P_	V_A_/V_P_	V_D_/V_P_	V_G_/V_P_	V_A_/V_P_
W12	0.503	0.536	0.049	0.585	0.554
EP	0.395	0.364	0.031	0.396	0.376
BMP	0.437	0.328	0.000	0.328	0.328

^a^*CADM* combining additive and dominance effects, *ADM* additive and dominance effects, *AM* additive effects only.

^b^*W12* body weight at the 12th week, *EP* eviscerating percentage, *BMP* breast muscle percentage.

Similar to the situations with chicken traits, the variance component values estimated from the three genomic models were different in pig traits (Table 2). For T1 and T3, the composite genetic variances estimated from the CADM model were highest among the three models. For T1, the ADM model did not capture the dominance genetic variance, and the variances estimated from the ADM and AM models were consistent. For T3, the ADM model captured the dominance genetic variance, but the cumulative genetic variance of additive and dominance effects was lower than the composite genetic variance from the CADM model. For T2, the composite genetic variance estimated from the CADM model was similar to the total genetic variance estimated by the ADM model, the ADM model captured the dominance genetic variance, and the additive variance estimated from the ADM model was similar to that from the AM model.

**Table 2:** Proportion of genetic variance based on the three genomic models^a^ for the pig genomic dataset^b^.

Trait	CADM	ADM			AM
	V_G*_/V_P_	V_A_/V_P_	V_D_/V_P_	V_G_/V_P_	V_A_/V_P_
T1	0.200	0.033	0.000	0.033	0.033
T2	0.276	0.263	0.015	0.278	0.264
T3	0.306	0.213	0.066	0.280	0.224

^a^*CADM* combining additive and dominance effects, *ADM* additive and dominance effects, *AM* additive effects only.

^b^The data concealed the actual trait names but used the symbols T1, T2, and T3.

### Genomic predictive ability

As shown in Table 3, in chickens, fivefold cross-validation revealed that the CADM model had the highest genomic predictive ability among the three models (more results of each of the replicates are shown in Supplementary File 2, Tables S1 and S2). Compared with the AM model, the genomic prediction accuracy was substantially improved for all three traits, with an average increase of 27.1%, and BMP (with the lowest heritability) had the largest increase, 46.1%. Compared with the AM model that considered additive effects only, the ADM model did not increase the genomic predictive ability. As shown in Table 4, the fivefold cross-validation for the pig dataset revealed that among the three genomic models, the CADM model had the highest genomic predictive ability for all three traits, similar to the results for the chicken data. Compared with the AM model, the accuracy of genomic prediction for the three traits increased by 57.9%, 5.4%, and 15.6%, respectively. T1 with the lowest heritability has the highest increase. The predictive ability of the ADM model did not improve or even slightly decreased compared with that of the AM model. In chicken dataset, the regression coefficients for the CADM and AM models were similar, ADM model had the largest bias for all the three traits (Table S3 in Supplementary File 2). In pig dataset, the regression coefficients for the CADM and AM models were similar for T2 and T3, CADM model had the less bias for T1 (Table S4 in Supplementary File 2).

**Table 3:** Genomic predictive ability^a^ for three models^b^ from a fivefold cross-validation analysis for the chicken dataset^c^.

Trait	CADM	ADM	AM
W12	0.585^A^ ± 0.012	0.504^B^ ± 0.014	0.502^B^ ± 0.014
EP	0.472^A^ ± 0.010	0.395^C^ ± 0.012	0.398^B^ ± 0.012
BMP	0.463^A^ ± 0.011	0.318^B^ ± 0.012	0.317^B^ ± 0.012

^a^Predictive ability was measured as the correlations between estimated genetic values (additive genetic values or additive + dominance genetic values) and adjusted phenotypes.

^b^*CADM* combining additive and dominance effects, *ADM* additive and dominance effects, *AM* additive effects only.

^c^*W12* body weight at the 12th week, *EP* eviscerating percentage, *BMP* breast muscle percentage.

^A–C^Within a row, estimates without a common superscript differ significantly (*P* < 0.01), according to bootstrap and Bonferroni’s methods test.

**Table 4:** Genomic predictive ability^a^ for three models^b^ from a fivefold cross-validation analysis for the pig dataset^c^.

Trait	CADM	ADM	AM
T1	0.562^A^ ± 0.006	0.356^B^ ± 0.007	0.356^B^ ± 0.007
T2	0.719^A^ ± 0.003	0.682^B^ ± 0.003	0.682^B^ ± 0.003
T3	0.688^A^ ± 0.004	0.588^C^ ± 0.004	0.595^B^ ± 0.004

^a^Predictive ability was measured as the correlations between estimated genetic values (additive genetic values or additive + dominance genetic values) and adjusted phenotypes.

^b^*CADM* combining additive and dominance effects, *ADM* additive and dominance effects, *AM* additive effects only.

^c^The data concealed the actual trait names but used the symbols T1, T2, and T3.

^A–C^Within a row, estimates without a common superscript differ significantly (*P* < 0.01), according to bootstrap and Bonferroni’s methods test.

## Discussion

### The novel CADM model

The phenotypic performance of a trait is affected not only by additive genetic effects but also by dominance and epistatic effects (Radoev et al. 2008). Dominance effects include incomplete dominance, complete dominance and overdominance, and these effects vary for different traits. Typical genomic prediction models with dominance genetic effects assume that dominance genetic effects of all loci have the same variance and directly use genomic markers to construct a dominance genetic relationship matrix between individuals using the same matrix for all traits (Alves et al. 2020; Liu and Chen 2018; Su et al. 2012). In the present study, the SNP loci are weighted according to the degree of deviation between heterozygous and homozygous loci, which differentiates the degrees of dominance effects on the trait between loci. The assumption of the CADM model is closer to the true distribution of dominance effects where dominance effects could be large for some QTLs, and small or no effect for the others. This could be one of the reasons that the CADM improves the accuracy of predictions. To the best of our knowledge, this is the first study of weighting dominance genetic effects for genomic prediction. In addition, the method included the dominance effects by integrating dominance and additive genetic effects into one component without increasing the number of unknowns to be estimated. Therefore, it is hypothesized that the model can improve the accuracy of predictions. As expected, based on the chicken and pig data examined here, our method greatly improved the genomic predictive ability.

In this study, overdominance was not considered in the CADM model. This may not completely match the actual situation. In fact, the CADM model is available for overdominance by relaxing the limitation (0 ≤ *C*_d_ ≤ 2) of heterozygous genotype coefficients. However, according to our experience, relaxation sometimes leads to problems with convergence or abnormal solutions caused by the uncertainty of the estimated degree of dominance. Lower MAF together with linkage disequilibrium will strongly affect the estimates. In this study, we used MAF ≥ 0.01 because it is popularly used threshold for editing marker genotype data in genomic prediction. For calculating difference between three genotypes of a locus, how to handle the low MAF loci is a challenge.

### Genomic predictive ability

Since the dominance effect is an important factor influencing phenotype, previous studies (Aliloo et al. 2016; Garcia-Baccino et al. 2019; Heidaritabar et al. 2016; Su et al. 2012; Vitezica et al. 2018) have used models containing the dominance effect to improve genomic predictive ability, but the improvements were very small. Su et al. (2012) studied the dominance effect of pig average daily gain and found that the genomic predictive ability increased from 0.319 to 0.330 after the dominance effect was included in the model. Garcia-Baccino et al. (2019) analyzed three growth traits in American Angus males and found that the model contained a dominance effect that did not affect genomic predictive ability. Aliloo et al. (2016) studied genomic predictions using models with dominance effects for four traits in Holstein and Jersey cattle and found that only fat yield in Holstein showed a slight increase in genomic predictive ability (from 0.270 to 0.273). Heidaritabar et al. (2016) studied eight traits in a population of laying hens and found that genomic predictive ability did not improve after including dominance effects in the model. In contrast to these studies, the present study proposed a novel approach that employs a modification of heterozygous genotype coefficients to integrate additive and dominance effects. The method was validated using chicken and pig datasets, and the average genomic predictive ability was improved by more than 20%.

For different traits, the dominance effects on phenotypic variation are different, so the combined additive and dominance relationship matrix (G_ad_). Some traits may be mainly affected by additive genetic effects, and some traits may be mainly affected by nonadditive genetic effects (Xu 2013). The CADM model could benefit for the traits in which dominance effects are largely different between different QTLs. Currently, breeding programs usually consider only additive genetic effects, i.e., breeding values. However, for many economically important traits, especially those related to fitness, which usually have low heritability, dominance effects may play a substantial role. For these traits, an improvement in the total genetic values of a population is important. For this purpose, the model proposed in this study would be a good alternative for genomic prediction of total genetic values. The genetic effect estimated from the CADM may be not suitable for breeding based on additive genetic effect, but can be used for optimizing the mating scheme. In commercial populations, such as chicken, pig, and cattle, it is important to make use of both additive genetic effect and heterosis. A matting design based on results from the CADM would be a good alternative to improve performance of a commercial population. Every method has its limitations, the CADM model has to calculate genotype matrix for each trait. In contrast, the AM and ADM models only need to calculate the genotype matrix once and can be used for all traits. The comparison with corrected phenotype is unfavorable for AM model for which the predictions do not include dominance effect. We include AM model in this study, because AM is a classic genomic prediction model.

## Conclusion

With our CADM model that combines additive and dominance genetic effects through defining heterozygous genotype coefficients, the abilities of genomic prediction are greatly improved. The results from this study indicate that dominant genetic variation is one of the important sources causing phenotypic variation, and the novel prediction model integrating dominance effects can significantly improve the accuracy of genomic prediction.

## Supplementary information

Supplemental File 1 - simulation for CADM

Supplemental File 2 - Tables
