# Supplementary material for: Including dominance effects in the prediction model through locus-specific weights on heterozygous genotypes can greatly improve genomic predictive abilities
Source: Heredity (Edinb). 2022 Feb 5;128(3):154–8. doi: 10.1038/s41437-022-00504-6 (PMC8897419; doi:10.1038/s41437-022-00504-6)
Supplement: Supplementary file 1 — Supplemental File 1 - simulation for CADM [file 41437_2022_504_MOESM1_ESM.docx]

***Supplemental Material - simulation for CADM***

**Including dominance effects in the prediction model through locus-specific weights on heterozygous genotypes can greatly improve genomic predictive abilities**

Tianfei Liu^1,2,3^, Chenglong Luo^1,2*^, Jie Ma^1,2^, Yan Wang^1,2^, Dingming Shu^1,2^, Hao Qu^1,2*^, Guosheng Su^3^

^*^Corresponding author

^1^State Key Laboratory of Livestock and Poultry Breeding, Institute of Animal Science, Guangdong Academy of Agricultural Sciences, Guangzhou 510640, China

^2^Guangdong Provincial Key Laboratory of Animal Breeding and Nutrition, Institute of Animal Science, Guangdong Academy of Agricultural Sciences, Guangzhou 510640, China

^3^Center for Quantitative Genetics and Genomics, Department of Molecular Biology and Genetics, Aarhus University, DK-8830 Tjele, Denmark

> ##########Supplemental Material #################

> ## a simple simulation for CADM model ##

> ####################################################

> #10 individuals and 15 SNPs

>

> #Additive and dominance effect

> #markerEffA=rnorm(15,0,1)

> markerEffA

[1] -0.25076920 1.41878221 1.24664560 -0.33044598 0.30021626 0.26794481 -0.47411025 1.22549659

[9] -0.37625275 -1.55312002 -1.04132232 -0.01892164 2.12337403 -0.56015589 1.24171300

> #markerEffD=runif(15,0,2)*markerEffA

> markerEffD

[1] -0.14844920 1.06736692 1.53654525 -0.18295004 0.30347799 0.22062846 -0.14630281 0.74110938

[9] -0.69104281 -2.86843495 -1.17146243 -0.00162358 1.02087253 -0.36338037 2.31657742

>

> #Original genotypes

> #genotypeOri=matrix(sample(c(0,1,2),150,replace = T),10,15)

> genotypeOri

[,1] [,2] [,3] [,4] [,5] [,6] [,7] [,8] [,9] [,10] [,11] [,12] [,13] [,14] [,15]

[1,] 1 2 0 0 0 0 0 0 0 1 2 0 0 0 0

[2,] 0 0 2 0 0 0 2 2 0 2 1 1 0 2 0

[3,] 2 2 2 2 1 1 1 2 0 1 0 1 0 2 2

[4,] 1 1 2 1 0 2 1 2 2 2 0 1 2 2 0

[5,] 0 0 0 0 0 0 2 0 1 2 2 1 2 1 0

[6,] 1 2 1 1 0 2 1 0 2 2 1 2 2 0 0

[7,] 0 2 0 1 1 0 1 2 0 0 2 0 2 2 0

[8,] 1 2 2 1 1 2 0 1 0 2 0 2 1 2 1

[9,] 2 1 0 2 1 2 0 1 2 1 2 2 1 0 1

[10,] 1 2 0 1 2 0 0 1 1 2 2 2 2 1 1

>

> #Heterozygous genotypes

> genotypeD=genotypeOri

> mychoose=genotypeOri==1

> genotypeD[!mychoose]=0

>

> #Ture genetic value

> TBVA=genotypeOri%*%markerEffA

> TBVD=genotypeD%*%markerEffD

>

> #Variances

> vara=var(TBVA);vara

[,1]

[1,] 11.49734

> vard=var(TBVD);vard

[,1]

[1,] 4.880516

> lamada=1

> vare=vara+vard;vare

[,1]

[1,] 16.37785

>

> #Phenotype

> #yOri=50+TBVA+TBVD+rnorm(10,0,sqrt(vare))

> yOri

[,1]

[1,] 46.48191

[2,] 53.08025

[3,] 56.33443

[4,] 51.12670

[5,] 54.40620

[6,] 54.50049

[7,] 51.29733

[8,] 73.12570

[9,] 54.04732

[10,] 59.57390

>

> #Weights on heterozygous genotypes

> genotypeAD=genotypeOri

> snpMean=array(0,dim=c(15,3))

> for (snp in 1:15) {

+ mychoose0=genotypeOri[,snp]==0

+ mychoose1=genotypeOri[,snp]==1

+ mychoose2=genotypeOri[,snp]==2

+

+ snpMean[snp,1]=mean(yOri[mychoose0])

+ snpMean[snp,2]=mean(yOri[mychoose1])

+ snpMean[snp,3]=mean(yOri[mychoose2])

+ }

> ##if mean(A1A1)>mean(A2A2), A1A1 and A2A2 are recoded as 2 and 0

> snpMeanOri=snpMean

> mychoose=snpMeanOri[,1]>snpMeanOri[,3]

> snpMean[mychoose,1]=snpMeanOri[mychoose,3]

> snpMean[mychoose,3]=snpMeanOri[mychoose,1]

> genotypeAD[,mychoose]=abs(genotypeAD[,mychoose]-2)

>

> ##Calculate d

> d=(snpMean[,2]-snpMean[,1])/(snpMean[,3]-snpMean[,1])*2

> mychooseNa=is.na(d)

> d[mychooseNa]=1

>

> ##The boundaries of 0 and 2 for d

> mychoose1=d>2

> d[mychoose1]=2

> mychoose2=d<0

> d[mychoose2]=0

> d

[1] 2.0000000 0.0000000 0.5096273 2.0000000 1.7719852 1.2868594 0.0000000 2.0000000 2.0000000

[10] 0.3125656 0.1788486 0.8487435 2.0000000 1.9989350 2.0000000

>

> ##Weighted heterozygous genotypes

> for (snp in 1:15) {

+ mychoose=genotypeAD[,snp]==1

+ genotypeAD[mychoose,snp]=d[snp]

+ }

>

> ##### Function of accuracy calculation >>>>>

> acc<-function(y,genotype,lamada,TBV){

+ saf=apply(genotype,2,sum)/20

+ M=genotype-2*saf

+ sum2pq=0

+ for (i in 1:10) {

+ sum2pq=sum2pq+2*saf[i]*(1-saf[i])

+ }

+ sum2pq

+ G=M%*%t(M)/sum2pq

+ X=array(1,10);XX=t(X)%*%X

+ Z=diag(1,10,10)

+ XZ=t(X)%*%Z;ZX=t(Z)%*%X

+ ZZ=t(Z)%*%Z+solve(G)*c(lamada)

+

+ lhs=rbind(cbind(XX,XZ),cbind(ZX,ZZ))

+ rhs=rbind(t(X)%*%y,t(Z)%*%y);rhs

+ beta=solve(lhs)%*%rhs;beta

+ gebv=beta[-1]

+ return(cor(gebv,TBV))

+ }

> ##<<<<< Function of accuracy calculation #####

>

> #Accuracy

> ##Calculate accuracy of AM model

> y=yOri

> genotype=genotypeOri

> lamada=vara/(vard+vare)

> TBV=TBVA

> acc_AM=acc(y,genotype,lamada,TBV)

>

> ##Calculate accuracy of CADM model

> y=yOri

> genotype=genotypeAD

> lamada=(vara+vard)/vare

> TBV=TBVA+TBVD

> acc_CADM=acc(y,genotype,lamada,TBV)

>

> #Results

> vara

[,1]

[1,] 11.49734

> vard

[,1]

[1,] 4.880516

> acc_AM

[,1]

[1,] 0.548723

> acc_CADM

[,1]

[1,] 0.7272813
