# Supplementary material for: Including dominance effects in the prediction model through locus-specific weights on heterozygous genotypes can greatly improve genomic predictive abilities
Source: Heredity (Edinb). 2022 Feb 5;128(3):154–8. doi: 10.1038/s41437-022-00504-6 (PMC8897419; doi:10.1038/s41437-022-00504-6)
Supplement: Supplementary file 2 — Supplemental File 2 - Tables [file 41437_2022_504_MOESM2_ESM.docx]

***Supplemental Material - Tables***

**Including dominance effects in the prediction model through locus-specific weights on heterozygous genotypes can greatly improve genomic predictive abilities**

Tianfei Liu^1,2,3^, Chenglong Luo^1,2*^, Jie Ma^1,2^, Yan Wang^1,2^, Dingming Shu^1,2^, Hao Qu^1,2*^, Guosheng Su^3^

^*^Corresponding author

^1^State Key Laboratory of Livestock and Poultry Breeding, Institute of Animal Science, Guangdong Academy of Agricultural Sciences, Guangzhou 510640, China

^2^Guangdong Provincial Key Laboratory of Animal Breeding and Nutrition, Institute of Animal Science, Guangdong Academy of Agricultural Sciences, Guangzhou 510640, China

^3^Center for Quantitative Genetics and Genomics, Department of Molecular Biology and Genetics, Aarhus University, DK-8830 Tjele, Denmark

**Table S1-1. Accuracy**^1^ **of genomic prediction for body weight at the 12^th^ week from a 5-fold cross-validation analysis for the chicken dataset.**

| Replicates | CADM^2^ | ADM | AM |
| --- | --- | --- | --- |
| 1 | 0.6050 | 0.5205 | 0.5301 |
| 2 | 0.5931 | 0.5065 | 0.5137 |
| 3 | 0.5651 | 0.4506 | 0.4703 |
| 4 | 0.5881 | 0.4866 | 0.4990 |
| 5 | 0.5689 | 0.4819 | 0.4997 |
| 6 | 0.5911 | 0.4811 | 0.5062 |
| 7 | 0.5774 | 0.4689 | 0.4825 |
| 8 | 0.5876 | 0.4972 | 0.5157 |
| 9 | 0.5863 | 0.4741 | 0.4877 |
| 10 | 0.5889 | 0.5038 | 0.5221 |

^1^ Accuracy was measured as the correlations between estimated genetic values (additive genetic values or additive + dominance genetic values) and adjusted phenotypes.

^2^ CADM: combining additive and dominance effects, ADM: additive and dominance effects, AM: additive effects only.

**Table S1-2. Accuracy**^1^ **of genomic prediction for eviscerating percentage from a 5-fold cross-validation analysis for the chicken dataset.**

| Replicates | CADM^2^ | ADM | AM |
| --- | --- | --- | --- |
| 1 | 0.4686 | 0.3731 | 0.3820 |
| 2 | 0.4745 | 0.3986 | 0.4066 |
| 3 | 0.4893 | 0.4022 | 0.4046 |
| 4 | 0.4571 | 0.3764 | 0.3835 |
| 5 | 0.4669 | 0.3783 | 0.3955 |
| 6 | 0.4877 | 0.4053 | 0.4128 |
| 7 | 0.4629 | 0.3764 | 0.3909 |
| 8 | 0.4715 | 0.3909 | 0.3998 |
| 9 | 0.4716 | 0.4020 | 0.4080 |
| 10 | 0.4622 | 0.3814 | 0.3896 |

^1^ Accuracy was measured as the correlations between estimated genetic values (additive genetic values or additive + dominance genetic values) and adjusted phenotypes.

^2^ CADM: combining additive and dominance effects, ADM: additive and dominance effects, AM: additive effects only.

**Table S1-3. Accuracy**^1^ **of genomic prediction for breast muscle percentage from a 5-fold cross-validation analysis for the chicken dataset.**

| Replicates | CADM^2^ | ADM | AM |
| --- | --- | --- | --- |
| 1 | 0.4539 | 0.2932 | 0.3128 |
| 2 | 0.4404 | 0.2863 | 0.2892 |
| 3 | 0.4795 | 0.3115 | 0.3274 |
| 4 | 0.4562 | 0.3161 | 0.3279 |
| 5 | 0.4826 | 0.3412 | 0.3453 |
| 6 | 0.4705 | 0.3143 | 0.3236 |
| 7 | 0.4572 | 0.3103 | 0.3120 |
| 8 | 0.4663 | 0.3031 | 0.3091 |
| 9 | 0.4537 | 0.2887 | 0.2955 |
| 10 | 0.4733 | 0.3233 | 0.3331 |

^1^ Accuracy was measured as the correlations between estimated genetic values (additive genetic values or additive + dominance genetic values) and adjusted phenotypes.

^2^ CADM: combining additive and dominance effects, ADM: additive and dominance effects, AM: additive effects only.

**Table S2-1. Accuracy**^1^  **of genomic prediction for T1 from a 5-fold cross-validation analysis for the pig dataset.**

| Replicates | CADM^2^ | ADM | AM |
| --- | --- | --- | --- |
| 1 | 0.5787 | 0.4131 | 0.3773 |
| 2 | 0.5565 | 0.4060 | 0.3612 |
| 3 | 0.5593 | 0.3754 | 0.3577 |
| 4 | 0.5564 | 0.3530 | 0.3437 |
| 5 | 0.5863 | 0.3879 | 0.3690 |
| 6 | 0.5504 | 0.3726 | 0.3376 |
| 7 | 0.5596 | 0.3656 | 0.3502 |
| 8 | 0.5622 | 0.3999 | 0.3645 |
| 9 | 0.5639 | 0.3955 | 0.3613 |
| 10 | 0.5452 | 0.3426 | 0.3408 |

^1^ Accuracy was measured as the correlations between estimated genetic values (additive genetic values or additive + dominance genetic values) and adjusted phenotypes.

^2^ CADM: combining additive and dominance effects, ADM: additive and dominance effects, AM: additive effects only.

**Table S2-2. Accuracy**^1^  **of genomic prediction for T2 from a 5-fold cross-validation analysis for the pig dataset.**

| Replicates | CADM^2^ | ADM | AM |
| --- | --- | --- | --- |
| 1 | 0.7204 | 0.7003 | 0.6831 |
| 2 | 0.7251 | 0.7002 | 0.6861 |
| 3 | 0.7164 | 0.6903 | 0.6800 |
| 4 | 0.7212 | 0.6990 | 0.6839 |
| 5 | 0.7185 | 0.6945 | 0.6814 |
| 6 | 0.7225 | 0.6982 | 0.6855 |
| 7 | 0.7163 | 0.6926 | 0.6798 |
| 8 | 0.7178 | 0.6939 | 0.6807 |
| 9 | 0.7198 | 0.7027 | 0.6833 |
| 10 | 0.7132 | 0.6929 | 0.6778 |

^1^ Accuracy was measured as the correlations between estimated genetic values (additive genetic values or additive + dominance genetic values) and adjusted phenotypes.

^2^ CADM: combining additive and dominance effects, ADM: additive and dominance effects, AM: additive effects only.

**Table S2-3. Accuracy**^1^  **of genomic prediction for T3 from a 5-fold cross-validation analysis for the pig dataset.**

| Replicates | CADM^2^ | ADM | AM |
| --- | --- | --- | --- |
| 1 | 0.6843 | 0.6804 | 0.5948 |
| 2 | 0.6879 | 0.6862 | 0.5947 |
| 3 | 0.6823 | 0.6716 | 0.5907 |
| 4 | 0.6727 | 0.6679 | 0.5834 |
| 5 | 0.6901 | 0.6848 | 0.5965 |
| 6 | 0.6919 | 0.6787 | 0.5978 |
| 7 | 0.6966 | 0.6956 | 0.6047 |
| 8 | 0.6923 | 0.6834 | 0.5980 |
| 9 | 0.6987 | 0.6885 | 0.6028 |
| 10 | 0.6782 | 0.6735 | 0.5883 |

^1^ Accuracy was measured as the correlations between estimated genetic values (additive genetic values or additive + dominance genetic values) and adjusted phenotypes.

^2^ CADM: combining additive and dominance effects, ADM: additive and dominance effects, AM: additive effects only.

**Table S3.** **Unbiasedness**^1^ **of genomic prediction for three models**^2^ **from a 5-fold cross-validation analysis for the chicken dataset** ^3^**.**

| **Trait** | **CADM** | **ADM** | **AM** |
| --- | --- | --- | --- |
| W12 | 1.075±0.028 | 0.561±0.015 | 1.076±0.029 |
| EP | 0.969±0.025 | 0.509±0.015 | 0.978±0.028 |
| BMP | 1.021±0.033 | 0.504±0.022 | 0.973±0.042 |

^1^ Unbiasedness was measured as the regressions between estimated genetic values (additive genetic values or additive + dominance genetic values) and adjusted phenotypes.

^2^ CADM: combining additive and dominance effects, ADM: additive and dominance effects, AM: additive effects only.

^3^ W12: body weight at the 12^th^ week, EP: eviscerating percentage, BMP: breast muscle percentage.

**Table S4. Unbiasedness**^1^ **of genomic prediction for three models**^2^ **from a 5-fold cross-validation analysis for the pig dataset** ^3^**.**

| **Trait** | **CADM** | **ADM** | **AM** |
| --- | --- | --- | --- |
| T1 | 2.132±0.03 | 2.342±0.051 | 4.665±0.101 |
| T2 | 1.351±0.008 | 0.671±0.004 | 1.338±0.008 |
| T3 | 1.588±0.011 | 0.838±0.007 | 1.67±0.014 |

^1^ Unbiasedness was measured as the regressions between estimated genetic values (additive genetic values or additive + dominance genetic values) and adjusted phenotypes.

^2^ CADM: combining additive and dominance effects, ADM: additive and dominance effects, AM: additive effects only.

^3^ The data concealed the actual trait names but used the symbols T1, T2, and T3.
